# Loneliness and academic performance mediates the relationship between fear of missing out and smartphone addiction among Iranian university students

**DOI:** 10.1186/s12888-022-04186-6

**Published:** 2022-08-12

**Authors:** Vahid Alinejad, Naser Parizad, Malakeh Yarmohammadi, Moloud Radfar

**Affiliations:** 1 Department of Epidemiology and Biostatistics, School of Medicine, Urmia University of Medical Sciences, Urmia, Iran; 2 Patient Safety Research Center, Clinical Research Institute, Urmia University of Medical Sciences, Urmia, Iran; 3 Department of critical care nursing, School of Nursing and Midwifery, Urmia University of Medical Sciences, Urmia, Iran; 4 Department of Psychiatric Nursing, School of Nursing and Midwifery, Urmia University of Medical Sciences, Urmia, Iran

**Keywords:** FoMO, Social network, Messaging applications, Smartphone addiction, Loneliness, Academic performance, Students

## Abstract

**Background:**

Fear of missing out (FoMO) can increase loneliness and smartphone addiction and decrease academic performance in university students. Most studies investigated the relationship between FoMO and smartphone addiction in developed countries, and no studies were found to examine this association in Iran. The mediating role of loneliness and academic performance in the relationship between FoMO and smartphone addiction is unclear. This study investigated the relationship between FoMO and smartphone addiction and the mediating role of loneliness and academic performance in this relationship in Iranian university students.

**Methods:**

In this cross-sectional study, 447 students from Urmia University of Medical Sciences were investigated. Data were collected using demographic questionnaires, Przybylski's FoMO scale, Pham and Taylor's academic performance questionnaire, Russell's loneliness scale, and Kwon's smartphone addiction scale. Data were analyzed using SPSS ver. 23 and SmartPLS ver. 2.

**Results:**

FoMO had a positive and direct association with smartphone addiction (β = 0.315, t-value = 5.152, *p* < 0.01). FoMO also had a positive and direct association with students’ loneliness (β = 0.432, t-value = 9.059, *p* < 0.01) and a negative and direct association with students' academic performance (β = -0.2602, t-value = 4.201, *p* < 0.01). FoMO indirectly associated with smartphone addiction through students' loneliness (β = 0.311, t-value = 5.075, *p* < 0.01), but academic performance was not mediator of smartphone addiction (β = 0.110, t-value = 1.807, *p* > 0.05). FoMO also indirectly correlated with academic performance through students' loneliness (β =—0.368, t-value = 6.377, *p* < 0.01).

**Conclusions:**

FoMO can be positively associated with students' smartphone addiction, and loneliness is an important mediator of this association. Since smartphone addiction could harm students' academic performance, thus, healthcare administrators should reduce students' loneliness and improve their academic performance by adopting practical strategies to help students to manage their time and control their smartphone use. Holding self-management skills classes, keeping students on schedule, turning off smartphone notifications, encouraging students to engage in sports, and participating in group and family activities will help manage FoMO and loneliness.

## Background

Smartphone addiction, sometimes referred to as "nomophobia" or " problematic smartphone use, " has recently attracted the attention of researchers [1]. Nowadays, smartphone addiction is not listed as addiction in the book Diagnostic and Statistical Manual of Mental Disorders, Fifth Edition (DSM-5) and the International Classification of Diseases Tenth Edition (ICD-10) [2]. However, smartphone addiction is one of the most significant challenges of the current century that can have serious consequences [1, 3]. Despite the numerous benefits of smartphones, their overuse can lead to problems and harm [4]. The negative effects of smartphone addiction can be physical, psychological, social, and behavioral [1, 4]. Some of the most important consequences are headache, hypertension, heart problems, eye problems, sleep disorders [1, 3], academic anxiety, depression, suicidal ideation, mental problems [1], and behavioral disorders such as fear of missing out (FoMO), textaphrenia, textiety, post-traumatic text disorder, or binge texting [1, 5].

Recent technologies can help students achieve their goals [4, 6]. However, excessive use of smartphones can be associated with distraction and reduced productivity among students and cause a decline in their academic performance [7]. Smartphone addiction should be considered much more seriously by medical students because it can endanger patients' safety [8]. The addiction rate of nursing, medical, and paramedical students is growing globally and in Iran[7–10]. Amiri et al. (2020) conducted a study in Iran and showed that smartphone addiction is 0.9 to 64.5% depending on the study population and measurement tools [11]. A review of the literature showed that students' addiction was high in South Korea with 47.83% [12], in the United States with 25.3% [13], in Spain with 22% [9], and in Saudi Arabia with 36.5% [9].

A literature review showed that smartphone addiction was associated with various factors such as depression, anxiety, insomnia, loneliness [1, 6, 8], academic failure [8, 14], and FoMO [15, 16]. Ezoe and Toda conducted a study and found a correlation between loneliness, internet addiction, and smartphone addiction in Japanese medical and nursing students [17]. Excessive use of smartphones among students can either assist their education [18] or cause distraction, waste students’ time, and negatively affect their performance [7]. Researchers have shown that FoMO can lead to daily behaviors such as excessive social networks use, using smartphones before going to bed, immediately after waking up, and during meals [19]. Most studies have investigated the relationship between FoMO and smartphone addiction in developed countries, and no studies have examined this association in Iran. The mediating role of loneliness and academic performance in the relationship between FoMO and smartphone addiction is unclear. Due to the critical role of smartphone addiction in medical, paramedical, and nursing students and its serious and irreversible consequences in care for patients, and to better understand this phenomenon, it is necessary to conduct research according to Iranian society's cultural and indigenous conditions. Therefore, the main purpose of this study was to investigate the direct and indirect association of variables on smartphone addiction in Iranian university students. To the best of our knowledge, this is the first study investigating the relationships between FoMO, loneliness, academic performance, and smartphone addiction among Iranian university students.

### Theoretical framework and development of hypotheses

#### Smartphone addiction

Smartphone addiction involves excessive cell phone use and is associated with functional disorders and symptoms observed in substance abuse, such as withdrawal and tolerance syndrome [20]. Smartphone addiction can have adverse effects on students' academic performance. Most students with this addiction suffer from sleep disorders, poor eating habits, lack of energy, obesity, and poor academic performance [21]. Several factors can lead students to smartphone addiction, including emotional problems, loneliness, poor academic performance [1, 3, 6, 8], and FoMO [19].

Smartphone addiction has been considered a disorder and conceptualized as an addictive behavior [22]. Indeed, smartphone addiction has been largely described as its continued use despite adverse effects, causing people to obsessively use their phones in improper situations such as during class, driving, or sleeping at night [23]. Attachment theory, developed by Bowlby (1969), has evolved into one of the leading theoretical frameworks for understanding smartphone addiction [24]. Although attachment theory was initially conceptualized to explain bonding connections between people, it has since been successfully involved in bonding relationships with objects such as a smartphone [22].

#### FoMO and academic performance

FoMO was conceptualized using self-determination theory (SDT) developed by Ryan and Deci (2000) [25] and used by Przybylski et al. (2013) to comprehend what causes FoMO [19]. Przybylski applied SDT to FoMO, suggesting that FoMO is a negative emotional state resulting from unmet social requirements. European psychologists define FoMO as a pervasive concern that other people may be gaining valuable experiences while one is missing them [19, 26]. One of the hallmarks of FoMO is the desire to be constantly connected to what others are doing. Przybylski believed that FoMO is a negative emotional state resulting from unmet social communication needs [19]. Students with FoMO overuse smartphones, and spending too much time on social networks disrupts their sleep patterns. They also suffer from academic failure due to stress and lack of sleep [27, 28]. Previous theory and research have found connections between FoMO, high anxiety levels, and impaired cognitive levels, which may associate with poor academic outcomes [29]. This relationship drives research to investigate the effect FoMO may have on academic performance [30] and how this effect can be explained. Thus, we devised our first hypothesis to test this link in our study.

### Hypothesis 1: FoMO is negatively related to academic performance

#### FoMO and loneliness

FoMO can facilitate loneliness in a person [15]. Previous conceptual models showed that FoMO had been associated with higher levels of loneliness and positively predicted it [15, 31]. An explanation for these interrelationships between social network use, FoMO, and loneliness is that people believe that their friends are involved in social events and are more successful based on their social network information. Therefore, they feel envious, lonely, and less connected to friends and suffer FoMO [16, 20].

### Hypothesis 2: FoMO is positively associated with loneliness

#### FoMO and smartphone addiction

Previous studies investigated the correlation between smartphone addiction and the impact of negative emotions, such as increasing FoMO, to evaluate factors that may contribute to social media addiction [32, 33]. Several studies showed a positive correlation between FoMO and smartphone addiction [20, 34, 35]. Research showed a moderating association between college students' FoMO and their use of smartphone addiction to fill their emotional and psychological deficiencies [19, 27]. The association between FoMO and smartphone addiction can be explained by the "Use and Satisfy" theory [36]. This theory posits that FoMO is derived from lacking basic psychological needs. FoMO lets individuals use smartphones to create and maintain social connections, carry out controllable interpersonal interactions, satisfy their psychological needs of relationship, independence, and competence, and then overuse them [37].

### Hypothesis 3: FoMO is positively associated with smartphone addiction

#### Academic performance and smartphone addiction

Academic performance is defined as how a learner achieves educational goals that are usually cognitive and about a particular topic [38]. Academic performance can be affected by various factors such as time management skills, smartphone addiction, FoMO, and social networks, and challenges students achieving their goals [21, 39]. Previous studies confirmed the negative association between problematic smartphone use and academic performance [14, 18, 21]. On the other hand, the positive effect of smartphone use was reported in students with high time management skills [21]. Students who cannot manage time will have poor academic performance. Students with high academic performance can manage their time and have their smartphone use under control [40].

Because this study coincided with the Covid 19 pandemic, students must use smartphones and the Internet more than usual due to online classes and examinations. This unique situation and the effort of students to enhance their academic performance could predispose them to smartphone addiction. The Uses and Gratifications theory states that people will seek the media when they get pleasure from the media [21]. Smartphones have become essential for students in their daily lives and studies, and they use smartphones to release stress, communicate with friends and family, and search for information. In addition, due to virtual and online education, university students may become more dependent on smartphones, ultimately leading to smartphone addiction [41]. Accordingly, academic performance could act as a mediator of problematic smartphone use by students. To the best of our knowledge, the mediating role of students' academic performance on smartphone addiction was not investigated. Thus, we hypothesized that there might be a positive relationship between academic performance and smartphone overuse.

### Hypothesis 4: Academic performance is positively associated with smartphone addiction

#### Loneliness and smartphone addiction

Loneliness is often defined based on a person's relationship with others, or specifically as an unpleasant experience occurring when a person's social relationships are disrupted in various ways [42]. Loneliness is a serious problem for school and university students. Lonely people are more prone to smartphone addiction [8] to compensate for the lack of offline relationships [42]. Although some studies showed no significant relationship between smartphone addiction and loneliness [10, 43], many previous studies reported a significant relationship between loneliness and smartphone addiction and its growing use [3, 8, 44].

Loneliness compels people to use a smartphone to build relationships and satisfy the feeling of belonging [15]; lonely people feel neglected, and they attract to repetitive behavior as a tool of mood enhancement, and their psychological dependency increases once the repetitive behavior is strengthened and often turns into an addiction [3]. People who experience loneliness try to cope with the bothersome feeling of loneliness by using smartphones excessively [8]. This study was conducted during pandemic lockdowns, which may worsen the students' feelings of loneliness by limiting social connections. Using smartphone-based technologies such as social networks (e.g., Instagram) and messaging applications (e.g., WhatsApp) could be a possible remedy to pandemic-induced social isolation [10, 44]. Based on the Compensatory Internet Use theory, when people are maladjusted in their life, they may use smartphones to escape negative emotions such as loneliness [45]. According to empirical evidence and the theory, we hypothesized that smartphone addiction among university students is predicted by loneliness. 

### Hypothesis 5: Loneliness is positively associated with smartphone addiction

#### Loneliness and academic performance

Because of the Covid 19 pandemic and social isolation, all individuals, particularly university students, experienced a high level of loneliness [46]. Loneliness is one of the psychosocial issues that result in academic procrastination. This academic failure could be explained by psychosocial syndemic theory, which states that the presence of more than one psychosocial problem can increase academic procrastination [47]. A previous study showed a significant positive relationship with moderate strength between loneliness and academic procrastination. A higher level of loneliness is associated with higher academic procrastination. [48].

### Hypothesis 6: Loneliness is negatively associated with academic performance

## Methods

### Research design and sampling

In this descriptive cross-sectional study, 447 students who met the inclusion criteria were recruited from the Urmia University of Medical Sciences. Students were selected from schools of medicine, pharmacy, dentistry, nursing and midwifery, and health and rehabilitation sciences. We used sample-to-item ratio criteria to determine sample size, and the ratio should not be less than 5-to-1 [49]. Because the number of questionnaire items is 88 (FoMO [10 items], loneliness [20 items], academic performance [48 items], and smartphone addiction [10 items]), therefore, we recruited 447 university students to cover the path analysis and test our mediation hypothesis. After determining the sample size, a proportional allocation was performed by stratified sampling method based on the schools of Urmia University of Medical Sciences, as follows:

First, the total statistical population of Urmia University of Medical Sciences students (3057 students) was determined and then divided by the number of requested samples (447 students).

$$\frac{\mathrm{n}}{\mathrm{N}}= \frac{447}{3057}=0.1462$$

Then, the obtained fraction (0.1462) was multiplied by the number of students from each category to determine how many students should be selected (Table 1).

**Table 1:** How to calculate the number of participants in different faculties

Faculty	Total students	Sample
Medical school	1318	192
School of dentistry	269	40
School of Pharmacy	220	32
Nursing and Midwifery school	510	75
School of Health	299	44
School of Paramedical Sciences	441	64
Total	3057	447

Inclusion criteria were: being students in one of the majors in Urmia University of Medical Sciences, willing to participate in the study, and having a smartphone. Exclusion criteria included unwillingness to stay in the study, incomplete completion of the questionnaires, and transferring to other academic centers.

### Procedure

After obtaining permission from the University's Ethics and Research Committee, an approval letter was presented to the dean of the faculties. The lead researcher explained the study's process, objectives, and sampling method to the dean of faculties. After obtaining the faculty's agreement, the list of students and their contact numbers were obtained from the education deputy. The lead researcher contacted the students through the faculty's education office based on the sample size calculated for each faculty. The lead researcher thoroughly explained how to complete the questionnaires and addressed their possible concerns and questions. It was also explained that participation in the study was voluntary, and they could leave the study at any time. Students were also assured that their information remained completely confidential. If they wished to participate in the study, informed consent forms were sent to them through the WhatsApp application, and they signed and returned it to the researcher before entering the study. After sampling was finished, the questionnaires were distributed to students online through class representatives. The students were asked to refer to the link (https://survey.porsline.ir/s/2qDd109/) and complete the online questionnaire. They were also requested to complete and return the questionnaire within 72 h. If students did not return the completed questionnaire within 72 h, they were asked again to complete and return the questionnaire. After the second request, if the students did not complete and send the questionnaire, they were excluded from the study. Out of 450 questionnaires, 447 questionnaires were completed and returned. Data collection took a month to be finished.

### Measures

Four questionnaires were used to collect data. Demographic information questionnaire included age, gender, marital status, residence status, number of semesters, college degrees, the field of study, rate of phone use per day, internet access rate, the purpose of smartphone use, type of application used, the number of text message per day, phone bill cost, and the number of daily calls.

Dr. Przybylski's FoMO scale consists of 10 questions on a five-point Likert scale (not at all true of me = 1, slightly true of me = 2, moderately true of me = 3, very true of me = 4, extremely true of me = 5); the whole scale range is 10 to 50, where higher scores indicate a higher level of FoMO [19]. In the study of Bayrami et al. (2019), the questionnaire was translated from English to Persian by a bilingual expert person using the forward and backward method; after translation, it was reviewed by ten faculty members, and validity was confirmed. Cronbach's alpha of the FoMO questionnaire was obtained at 0.87 [50].

Pham and Taylor's academic performance questionnaire consisting of 48 questions was used to assess academic performance [51]. It assesses academic performance from various components: self-efficacy, emotional impact, planning, lack of outcome control, and motivation. The questionnaire is scored on a 5-point Likert scale (none = 1 and very high = 5). The score of questions related to the emotional impact and lack of outcome control dimensions should be reversed and then added to the score of other dimensions to calculate the total academic performance score. Pham and Taylor reported this questionnaire's internal and external validity as 0.83 and 0.79, respectively [51]. This questionnaire was evaluated in the Dortaj study (2004), and professors confirmed the questionnaire's content validity. Cronbach's alpha coefficient was used to evaluate the reliability of the questionnaire. Cronbach's alpha of self-efficacy, emotional impact, planning, lack of outcome control, motivation, and the total scale were 0.92, 0.73, 0.93, 0.64, 0.73, and 0.74, respectively [52].

Russell et al. (1980) developed Russell's loneliness scale from the University of California, which is a revised University of California, Los Angeles (UCLA) main scale consisting of 20 questions [53]. The questionnaire has 20 questions (10 negatives and 10 positives), scored from 1 to 4, respectively, based on a 4-point Likert scale (never, rarely, sometimes, always). The minimum and maximum scores are between 20 to 80, and the average score is 50. A score higher than the average indicates severe loneliness. This questionnaire was administered to four groups of students, nurses, teachers, and the elderly in different ways, such as through self-report and interview, and the alpha range was obtained from 0.89 to 0.94 [53]. The structural validity and reliability of the questionnaire were confirmed by Hojat (1982) on Iranian students, and the reliability of the questionnaire was confirmed through Cronbach's alpha split-half method (α = 0.89) [54].

Kwon et al.'s Smartphone Addiction Scale-Short Version (SAS-SV) consists of 10 questions, and its purpose is to assess the level of students' smartphone addiction [55] based on a 6-point Likert scale (strongly disagree = 1 to strongly agree = 6). The overall score on this scale is 10 to 60; the highest score indicates the highest level of smartphone addiction. Kwon et al. (2013) reviewed and approved this questionnaire regarding content validity index, concurrent validity, and internal stability reliability. The total reliability of the questionnaire was calculated at 0.911, using Cronbach's alpha [55]. Fallahtafti et al. (2020) translated this questionnaire into Persian and examined its validity and reliability in Iran. They described it as credible and reliable in Persian. The reliability of the questionnaire was estimated at 0.82 using Cronbach's alpha [56].

### Data analysis

SPSS ver. 23 (IBM Corp., Armonk, N.Y., USA) and SmartPLS 2.0 (Beta) were used to analyze the data. Quantitative variables were reported as mean ± standard deviation, and qualitative variables were reported as number and percentage. The Fornell-Larcker method was used to evaluate the model's discriminant validity, and to confirm the model's reliability, the internal consistency method (Cronbach's alpha coefficient) was used. Path coefficients (β) and t-coefficients were used to investigate the conceptual model. We also used the nonparametric percentile Bootstrap method to test the mediating role of variables. A P-value less than 0.05 was considered significant.

## Results

The mean age of 447 students participating in the study was 23.78 ± 4.038 years; 97 (21.7%) students were married, and 350 (78.3%) were single. Also, 170 (38%) were male, and 277 (62%) were female; 254 (54.8%) had a bachelor's degree, 70 (15.7%) master's degree, 95 (21.3%) doctor of medicine (MD), and 31 (6.9%) had a doctor of philosophy (Ph.D.); 242 (54.1%) lived in a dormitory, 159 (35.6%) were native students, and 46 (10.3%) were not native but did not live in a dormitory. Of the 447 students participating in the study, 80 students (17.9%) worked 2 h a day with their smartphones, 95 (21.3%) 4 h, 153 (34.2%) 6 h, 83 (18.6%) 8 h, 27 (6%) 10 h a day, and 9 (2%) worked 12 h a day with their smartphones; 161 (36%) students used their smartphones for non-academic purposes, and 286 (64%) used their smartphones for academic purposes. Also, 95 (21.3%) students used the Telegram application, 232 (51.9%) Instagram, 74 (16.6%) WhatsApp, and the rest of the students used other mass media applications. Finally, 276 (61.7%) students had less than 40 text messages per day, and 171 (38.3%) students sent more than or equal to 40 text messages per day (Table 2). The results showed that Cronbach's alpha values for all variables were above 0.7. It can be inferred that the model has good internal reliability (Table 3). According to Fornell-Larcker's criterion, the discriminant validity of the model is acceptable (Table 4).

**Table 2:** Demographic characteristic of participants in the study

Variables		Number	Percent
**Gender**	Male	170	38.0
	Female	277	62.0
**Marital status**	Married	97	21.7
	Single	350	78.3
**Residence status**	Dormitory	242	54.1
	Non-dormitory	46	10.3
	Native	159	35.6
**Educational status**	Bachelor's degree	254	54.8
	Master's degree	70	15.7
	PhD	31	6.9
	MD	95	21.3
**Time spent on smartphones (daily)**	2 h	80	17.9
	4 h	95	21.3
	6 h	153	34.2
	8 h	83	18.6
	10 h	27	6.0
	12 h	9	2.0
**Purpose of smartphone use**	Academic purpose	286	64.0
	non-academic purpose	161	36.0
**Type of applications used**	Telegram	95	21.3
	Instagram	232	51.9
	WhatsApp	74	16.6
	Others	46	10.3
**Text message rates (daily)**	< 40	276	61.7
	≥ 40	171	38.3

**Table 3:** Internal consistency reliability coefficients (Cronbach's alpha)

Variables	Cronbach's alpha coefficients
FOMO ^*^	0.865
Academic performance	0.948
Loneliness	0.916
Smartphone addiction	0.911

^*^ Fear of missing out

**Table 4:** Fornell and Larker method (discriminant validity)

Variables	FOMO ^*^	Academic performance	Loneliness	Smartphone addiction
FOMO ^*^	1			
Academic performance	- 0.4189	1		
Loneliness	0.4316	- 0.4801	1	
Smartphone addiction	0.4035	- 0.1713	0.3941	1

^*^ Fear of missing out

### General conceptual model

**Fig. 1:**
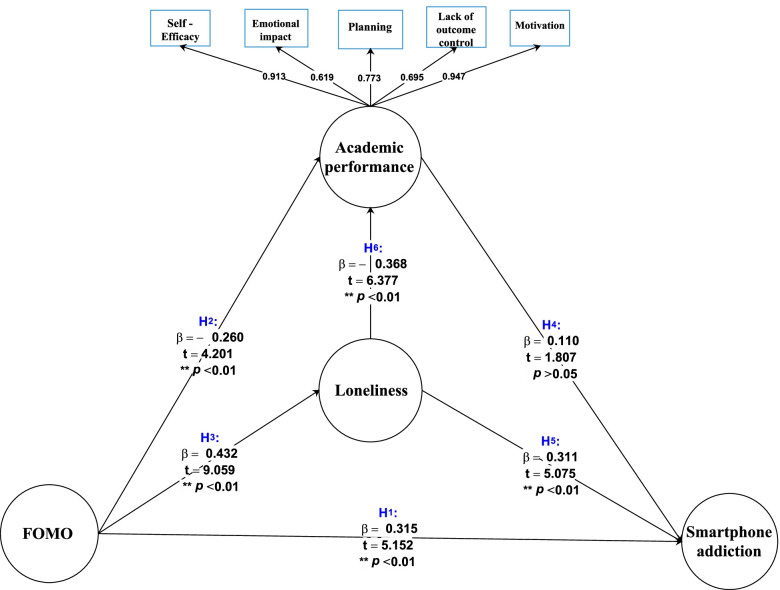
The conceptual model with beta coefficient and "t" values of path between study variables

The proposed conceptual model was evaluated after reviewing and testing the research hypotheses. The results are presented in two parts: path coefficients (β) and t-statistics. The results of this model are shown in Fig. 1 and Table 5. The path coefficient (β) indicates the direct association of an independent variable with the dependent variable. If the path coefficients between the variables are greater than 0.6, the predictor association of the latent variable with the dependent variable is strong. If this value is 0.3 to 0.6, the association is moderate, and if it is less than 0.3, it is considered weak. The parceling technique was used to investigate the three latent constructs of FOMO, loneliness, and smartphone addiction to improve the quality of indicators and model fit. Since these three scales contained a large number of items, the all-item-parcel approach was used to aggregate all items within a given scale and used this scale-composite score as the sole indicator of the target construct [57].

**Table 5:** Results of structural equation analysis for the general conceptual model

**Hypothesis**	*β*	*t*	*Sig*	*Result*
**H1:** FoMO → Smartphone addiction	0.315	5.152	< 0.01	Confirmed
**H2:** FoMO → Academic performance	- 0.260	4.201	< 0.01	Confirmed
**H3:** FoMO → Loneliness	0.432	9.059	< 0.01	Confirmed
**H4:** Academic performance → Smartphone addiction	0.110	1.807	> 0.05	Rejected
**H5:** Loneliness → Smartphone addiction	0.311	5.075	< 0.01	Confirmed
**H6:** Loneliness → Academic performance	- 0.368	6.377	< 0.01	Confirmed

According to Fig. 1: FoMO had a positive and moderate relationship with smartphone addiction (β = 0.315). FoMO had a positive and moderate association with loneliness (β = 0.432), and loneliness has a positive and moderate association with smartphone addiction (β = 0.311). FoMO had a negative and weak relationship with academic performance (β =—0.260), and the academic performance had a positive and weak relationship with smartphone addiction (β = 0.110). FoMO had a positive and moderate association with loneliness (β = 0.432), loneliness had a negative and moderate relationship with academic performance (β =—0.368), and the academic performance had a positive and weak relationship with smartphone addiction (β = 0.110) (Table 5) (Fig. 1).

**Fig. 2:**
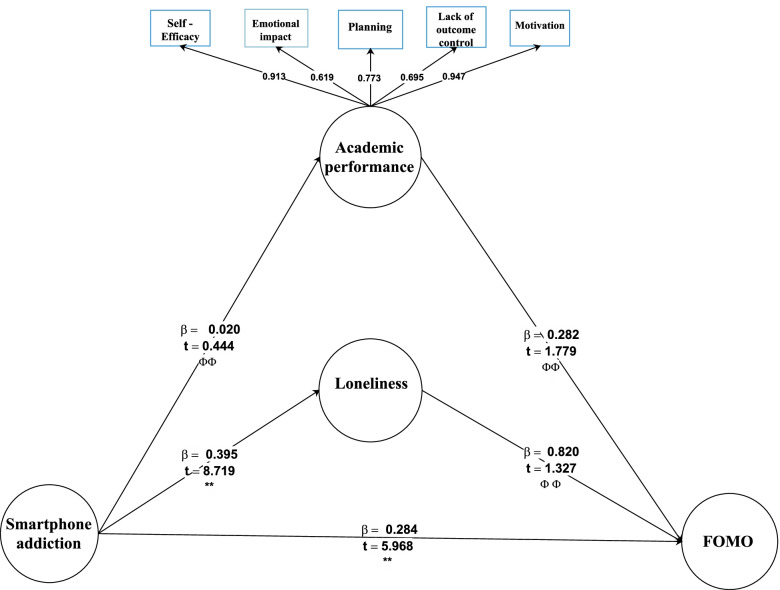
The reverse model

As shown in Fig. 2, the t-values of the relationship between the FoMO on smartphone addiction, FoMO on academic performance, FoMO on loneliness, loneliness on smartphone addiction, and academic performance are greater than the limited value of 1.96. Thus, it can be concluded that the mentioned variables have a significant relationship with each other with at least 95% confidence. Ultimately, the relationship between academic performance and smartphone addiction is not significant because the t value is less than 1.96 (Fig. 2).

The resultsshow the values of the path coefficient and the t-value at the 95% confidence level based on figure findings for the overall conceptual model (Table 4). In this study, there were two mediating variables, and the indirect relationships of the variables were investigated using the bootstrap method.

Table 6 shows the Multiple mediation test of indirect associations between variables using the Bootstrap method. Bootstrap test results showed that the upper and lower limits of the indirect relationship between FoMO and smartphone addiction through loneliness do not include zero, which means that this indirect relationship is significant (the mediator role of loneliness is confirmed). The upper and lower limits of the indirect association between FoMO and smartphone addiction through academic performance include zero, which means that this indirect association is not significant (the mediator role of academic performance was not confirmed). The upper and lower limits of the indirect relationship between FoMO and academic performance through loneliness do not include zero, which means that this indirect relationship is significant (the mediator role of loneliness is confirmed) (Table 6).

**Table 6:** Indirect Effects and Bootstrapping Results with all Paths (Multiple mediation analysis)

**Path**	*Indirect effect*	*Bootstrap standard errors*	*95% Bootstrapped Confidence Interval*	
			*Lower*	*Upper*
FoMO to smartphone addiction through **loneliness**	0.1549	0.0361	0.0882	0.2298
FoMO to smartphone addiction through **academic performance**	-0.0086	0.0277	-0.0636	0.0477
FoMO to academic performance through **loneliness**	-0.5080	0.0895	-0.6912	-.0.3406

Five observed variables and four latent variables, namely, FoMO, smartphone addiction, academic performance, and loneliness, were included in the hypothesized model. The measurement model had a good fitness as indicated by x^2^ = 28,479.31, df = 3648, *P* = 0.0001, RMSEA = 0.12; NNFI = 0.90; IFI = 0.89; GFI = 0.91 and the CFI = 0.92. Also, all the factor loadings for the indicators on the latent variables were significant (*P* < 0.001). It means that the indicators of all the latent variables represent them well.

### Reversed model

We checked the reverse model to confirm the causal relationships between latent variables. The paths between latent variables were assumed reverse to construct the reverse model. The results showed that some path coefficients between latent variables were not statistically significant, and the fit indices of the reverse model were unsatisfactory (P (RMSEA) > 0.05). Thus, the reverse model was not acceptable (Fig. 2).

## Discussion

FoMO causes anxiety and depression due to repetitive negative thoughts about disconnecting from the community and paves the way for smartphone addiction [20]. The results showed that FoMO had a direct and positive relationship with students' smartphone addiction and an indirect and negative relationship with students' academic performance through loneliness. FoMO is also indirectly related to smartphone addiction through students' academic performance, and loneliness. FoMO may lead to concerns that the student may miss an opportunity for social interaction, a new experience, or a memorable event and developmental and behavioral disorders [19, 26]. FoMO has been recently attributed to many negative psychological and behavioral symptoms [1, 4]. Smartphone addiction is one of these negative behaviors that can affect all aspects of a person's life [34, 35]. Numerous studies have confirmed the direct [20, 34, 35] or mediating relationship between FoMO and smartphone addiction [58, 59].

FoMO can associate with students' academic performance [27, 28]. In this study, FoMO was directly and indirectly associated with student performance. Qutishat et al.(2019) reported a negative impact of FoMO on students' academic performance at Sultan Qaboos University in Oman, which is in line with this study's results [60]. Students with FoMO increase their online social interactions [61], use the internet or cyberspace pathologically [26], and have sleep deprivation [27, 28], or FoMO may worsen students' anxiety and depression [21], which eventually leads to their academic failure.

FoMO had a direct and positive relationship with loneliness. A positive association between FoMO and loneliness was reported in several studies [15, 42]. Students have FoMO when they feel that their friends are interacting more on social media, increasing interaction with others [61]. When these students do not receive a response on social networks, the situation worsens; they feel lonelier than before, and sometimes they suffer stress and depression [62]. One explanation for this depression is that FoMO activates the idea that students' friends are getting engaged in some fun event and are more fortunate, which then evokes feelings of loneliness, and they suffer the fear of abandonment [16, 58]. Hunt et al. (2018) showed that limiting the use of virtual networks can positively and directly affect reducing loneliness and depression [62].

Our 4^th^ hypothesis was confirmed, and there was a positive and very weak relationship between students' academic performance and smartphone addiction, which was against the results of previous studies [3, 14]. However, this relationship was insignificant, and academic performance is not considered a mediator in this study. Being our study concurrent with the covid-19 pandemic, holding classes online, having university students spend a lot of time on the internet, and using their smartphones for educational purposes are plausible reasons that can justify our findings [15, 63]. Another possible reason is that students with good academic performance may have better time management and use their smartphones mostly to study their lessons or do tasks related to their homework assignments [18]. Khan et al. [21] found a strong and negative relationship between smartphone overuse and time management. Students with good academic performance had good time management, managed their time, used the internet and smartphones for educational purposes, and minimized virtual networks [21].

This study showed a positive and moderate relationship between loneliness and smartphone addiction, i.e., the more students felt lonely, the more they were drawn to smartphones, which was consistent with a recent study [64]. One of the most important reasons for smartphone addiction by students is escaping from loneliness [6]. On the contrary, Pittman et al. (2016) found that participants who used image-sharing platforms such as Instagram did not feel lonely but rather happy. They also reported no relationship between loneliness and text messaging platforms like Twitter [42]. A plausible reason for this could be students' place of study and majors. In their study, students studying in journalism and business majors had less stress than nursing, medical and para-medical students. They conducted the research in a developed country with many entertainments and few problems for university students.

The results indicated that loneliness had a negative and direct relationship with academic performance; it also acted as a mediator and enhanced the negative relationship between FoMO and academic performance. Hence, our 6^th^ hypothesis was confirmed. In line with our findings, Mahapatra (2019) reported loneliness as the primary antecedent for smartphone addiction and poor academic performance as its significant negative consequences [3]. A study conducted in Indonesia showed a weak but significant and positive association between loneliness and academic procrastination among psychology students [48]. This occurs because people are experiencing isolation during pandemics.

Moreover, interpersonal people with loneliness tend to have negative attitudes toward others, low self-esteem, and a lack of social capabilities. This result in social rejection that can cause people to feel lonely. Avoiding social interaction leads to academic procrastination or failure [47].

### Study limitations

The use of social networking applications such as Facebook and Twitter are not discussed separately from messaging applications such as Telegram and WhatsApp, and these two types of applications can have opposite effects [15]. Therefore, it is recommended to conduct a study to examine the impact of each application separately. Most participants were female and single, which could affect the study results [1, 20]. Other limitations of this study that could lead to smartphone addiction and high Internet use and challenge the interpretation of the results are that the study coincided with the COVID-19 pandemic, the lockdown of cities, decreased social presence and online education, lack of sports and recreational activities. Another limitation was the reduction in the quality of education and, consequently, the decline in students' academic performance during the pandemic, which directly impacted the results of this study. Similar studies are recommended after stabilizing the pandemic conditions to confirm the current results. Another study limitation is the cross-sectional design and convenience sampling, which includes a small portion of the Iranian student community at the Urmia University of Medical Sciences and failed to represent all Iranian university students. The self-report questionnaire that can lead to subjective biases is another study limitation that should be considered.

## Conclusion

The results showed that FoMO has a direct and positive relationship with smartphone addiction in university students. Also, loneliness is an important mediator of this relationship. Loneliness has an indirect and negative association with academic performance, and the association of loneliness with smartphone addiction is greater than its relationship with academic performance. Healthcare managers should reduce students' FoMO and loneliness and improve their academic performance by adopting practical strategies to help students manage their time and control their smartphone use. Accordingly, students can overcome their problems and achieve their goals. Holding self-management skills classes, keeping students on schedule, turning off smartphone notifications, encouraging students to engage in sports, and participating in group and family activities will help to manage FoMO and loneliness. Interruptions and distractions caused by using smartphones in clinical settings pose potential risks to patient safety. Therefore, it is essential to evaluate the use of smartphones at work and encourage graduate students and medical staff to assess their behaviors and help them understand the potential dangers. Thus, it is recommended to set the rules to regulate the use of smartphones during the clinical activities of staff and students.

## Data Availability

The datasets generated during and/or analyzed during the current study are available from the corresponding author on reasonable request.

## Abbreviations

FoMO: Fear of missing out
DSM-5: Diagnostic and Statistical Manual of Mental Disorders, Fifth Edition
ICD-10: International Classification of Diseases Tenth Edition
SDT: Self-determination theory
UCLA: University of California, Los Angeles
SAS-SV: Smartphone Addiction Scale- Short Version
SPSS: Statistical Package for the Social Sciences
MD: Doctor of Medicine
Ph.D.: Doctor of Philosophy
